# Prevalence rate of primary osteoporosis in China: a meta-analysis

**DOI:** 10.1186/s12889-024-18932-w

**Published:** 2024-06-06

**Authors:** Fang Fei Lyu, Vimala Ramoo, Ping Lei Chui, Chong Guan Ng, Yuanyuan Zhang

**Affiliations:** 1 School of Nursing, Yunnan University of Chinese Medicine, Kunming, Yunnan 650500 China; 2 Department of Nursing Science, Faculty of Medicine, Universiti Malaya, Kuala Lumpur, 50603 Malaysia; 3 Department of Psychological Medicine, Faculty of Medicine, Universiti Malaya, Kuala Lumpur, 50603 Malaysia; 4 School of Medical and Health Engineering, Changzhou University, Changzhou, Jiangsu 213000 China

**Keywords:** Primary osteoporosis, Prevalence, Meta-analysis, China

## Abstract

**Background:**

Primary osteoporosis (POP) is recognized as a “silent disease” and often ignored. This meta-analysis aimed to determine the prevalence of POP in the Chinese population over the past 20 years to raise awareness of the disease’s epidemiology, which is hoped to help prevent and treat the condition better.

**Methods:**

Eight English and three Chinese language databases were searched systematically from January 2002 to December 2023. Relevant data were analysed using Stata 16.0. Meta-regression and subgroup analyses were performed to explore causes of heterogeneity. A funnel plot was further drawn in combination with Egger and Begg tests to determine publication bias.

**Results:**

A total of 45 studies (241,813 participants) were included. The meta-analysis revealed that the overall prevalence of POP in the Chinese population was 18.2% (95% CI: 14.7–21.7%), showing a positive correlation with age. Specifically, prevalence rates were 23.4% (18.3–28.5%) in women and 11.5% (9.1–13.9%) in men. A notable increase was observed within the span of 20 years (16.9% before 2010 and 20.3% in 2011–2020). Notably, regional variations were observed, with southern China reporting a lower prevalence of 16.4% compared to 20.2% in northern China. Meta-regression suggested that sample size significantly influenced the estimation of point prevalence (*P* = 0.037).

**Conclusions:**

Over the past two decades, there has been an increase in the prevalence of POP within the Chinese population. The growing prevalence of older individuals and women further highlights the urgency for tailored disease prevention and control measures.

**Supplementary Information:**

The online version contains supplementary material available at 10.1186/s12889-024-18932-w.

## Background

Osteoporosis is divided into primary osteoporosis (POP) and secondary osteoporosis. POP is a systemic metabolic disease characterized by bone loss, degradation of bone microstructure, increased bone fragility, and susceptibility to fractures [1]. POP is further categorized into postmenopausal osteoporosis (PMOP), senile osteoporosis, and idiopathic osteoporosis. PMOP predominantly affects postmenopausal women, while senile osteoporosis is closely associated with aging. Idiopathic osteoporosis occurs in children and young adults, characterized by an unknown etiology [2].

Today, POP is the most common form of osteoporosis due to natural aging [3, 4]. The latest seventh national population census of China [5] showed that the country has the largest absolute number of older person in the world, at 260 million and 190 million aged above 60 and 65, respectively. Due to this large number of older people, POP poses a serious social problem among the Chinese population [6]. However, in China, there is a high prevalence of osteoporosis with low awareness, only 7% of patients over 50 having awareness [7]. The complications associated with POP include pain, fractures, disability, and reduced quality of life [8]. Fractures caused by POP, in particular, not only affect the patient’s quality of life but also cause serious burdens to families and the healthcare system [9, 10]. Insufficiently addressed, POP and its consequential fractures pose an imminent threat of imposing substantial medical and economic strains on China’s burgeoning aging population [11].

In recent years, nationwide multicenter studies have been conducted to determine the incidence of osteoporosis in China [12, 13], and the prevalence of osteoporosis has been summarized through systematic reviews and meta-analyses [14, 15]. However, these studies have not differentiated between primary and secondary osteoporosis. In addition, recent scholarly attention has been directed towards the age-standardized prevalence of osteoporosis among middle-aged and older individuals [16, 17]. Nonetheless, according to the classification of POP, it extends beyond the confines of the middle-aged and older population. A nuanced examination of POP prevalence across diverse age strata, geographical locales, and gender demographics within China emerges as imperative for fostering heightened awareness and targeted interventions. Therefore, this study systematically examined the prevalence of POP across all age groups in Chinese adults over the last two decades by comprehensively retrieving relevant literature.

## Materials and methods

This review was conducted in accordance with the Preferred Reporting Items for Systematic Reviews and Meta-Analyses Guidelines [18]. A review of the PROSPERO International Prospective Register of Systematic Reviews revealed no systematic reviews of the prevalence rate of POP in China.

### Search strategy

The electronic PubMed, Web of Science, Cochrane Library, Cumulative Index to Nursing and Allied Health Literature (CINAHL), MEDLINE, EMBASE, OVID, Scopus, China National Knowledge Infrastructure (CNKI) (Chinese), Wan Fang (Chinese), and China Science and Technology Journal Database (VIP) (Chinese) databases were used to search relevant studies published from January 1, 2002, to December 31, 2023. The search was supplemented with the “snowball” method for document tracing, along with manual retrieval of articles from preprint servers to access “grey” literature. Subject terms and free words were used in combination as search terms, including “osteoporosis” or “osteopenia” or “OP” or “primary osteoporosis” or “POP” or “bone mineral density” or “brittle bone disease” and “prevalence” or “epidemiology” or “morbidity” or “incidence” and “China” or “Chinese”. The language of publication was limited to English and Chinese.

### Literature inclusion and exclusion criteria

The following inclusion criteria were adopted:

(i) Study type: Cross-sectional studies.
(ii) Measurement of POP: Studies utilized dual-energy X-ray absorptiometry (DXA) for the measurement of POP.
(iii) Diagnostic criteria for POP: Studies adhered to the diagnostic criteria outlined by the World Health Organization (WHO), which defines POP as a bone mineral density (BMD) 2.5 standard deviations below the average value, indicated by a T-score of < -2.5 SD.
(iv) Participation: Studies involving populations aged ≥ 20 years,  encompassing young adults, postmenopausal women, middle-aged, and older individuals, drawn mainly from community settings. In addition, population with certain diseases (such as hyperthyroidism, hypogonadism, diabetes, rheumatoid arthritis, systemic lupus erythematosus, multiple myeloma, chronic diarrhea, lymphoma, malabsorption, stroke, chronic heart, lung or kidney diseases, etc.), and those taking medications known to affect bone metabolism (including glucocorticoid, antiepileptic drugs, antitumor chemotherapeutics and excessive thyroid hormone, etc.) were explicitly excluded in the original articles.
(v) Outcomes: Prevalence of POP or osteoporosis. The prevalence rates were reported with age stratification in five-year increments or in multiples of five years (e.g., 20–24 years, 25–29 years, or 20–29 years, and 30–39 years).

The exclusion criteria were as follows:

(i) Studies conducted in a population in which special groups or occupations were excluded (e.g., hospitalized patients and a population working in an environment with lead, cadmium, and aluminum).
(ii) Reviews, commentaries, and case reports.
(iii) Studies with incomplete data, and sample sizes of less than 100 participants.

### Literature screening and data extraction

Two reviewers, both trained in systematic review methodologies, conducted a comprehensive search of databases for relevant articles. Independently, they conducted preliminary screenings of titles and abstracts based on predetermined inclusion and exclusion criteria. Articles meeting the inclusion criteria underwent full-text review. Any discrepancies between the researchers were resolved through discussion or, if necessary, by the collective arbitration of the research group.

For each included study, two reviewers independently extracted the following information: (i) Basic Information: This included details such as the first author, year of publication, period of data collection, geographic area (northern or southern), age range, sample size, and the instruments and positions used for BMD measurement. (ii) Calculated Prevalence: This encompassed the number of POP cases and participants in each age-specific group. In cases where specific information was missing, the authors were contacted for clarification. (iii) Assessment of Study Quality: Relevant elements for assessing the quality of the study were also extracted. Any discrepancies between the two reviewers were resolved by a third reviewer.

### Quality assessment

The quality assessment of each study considered for further review utilized the quality assessment criteria for cross-sectional studies recommended by the Agency for Healthcare Research and Quality (AHRQ) [19]. These criteria consisted of eleven assessment points, with responses categorized as ‘Yes’ (one point), ‘No’, or ‘Unclear’ (both denoted as zero points). Each study could attain a maximum score of 11 points. Studies accumulating ≤ 5 points were deemed to be of low quality [20].

### Data analysis and synthesis

The meta-analysis was conducted using Stata 16.0 software. Firstly, heterogeneity analyses and model selection were performed. A significance level of *p* < 0.05 and *I*^2^ > 50% indicated substantial heterogeneity, leading to the selection of a random effects model (REM). Conversely, if *p* > 0.05 and *I*^2^ < 50%, suggesting lesser heterogeneity, a fixed effects model (FEM) was chosen. Secondly, subgroup analyses were conducted based on various factors, including period of data collection (∼ 2010 and 2011–2020), geographic area (South and North China), gender (men and women), BMD measurement positions (included lumbar spine and other), and age groups (20–29, 30–39, 40–49, 50–59, 60–69, 70–79, ≥ 80 years), both collectively and separately for each gender. Additionally, meta-regression was utilized to explore potential sources of heterogeneity. Thirdly, the impact of individual studies and the stability of the results were assessed using sensitivity analysis. Finally, funnel plots were generated and Egger and Begg tests were conducted to identify publication bias.

## Results

### Study selection

**Fig. 1:**
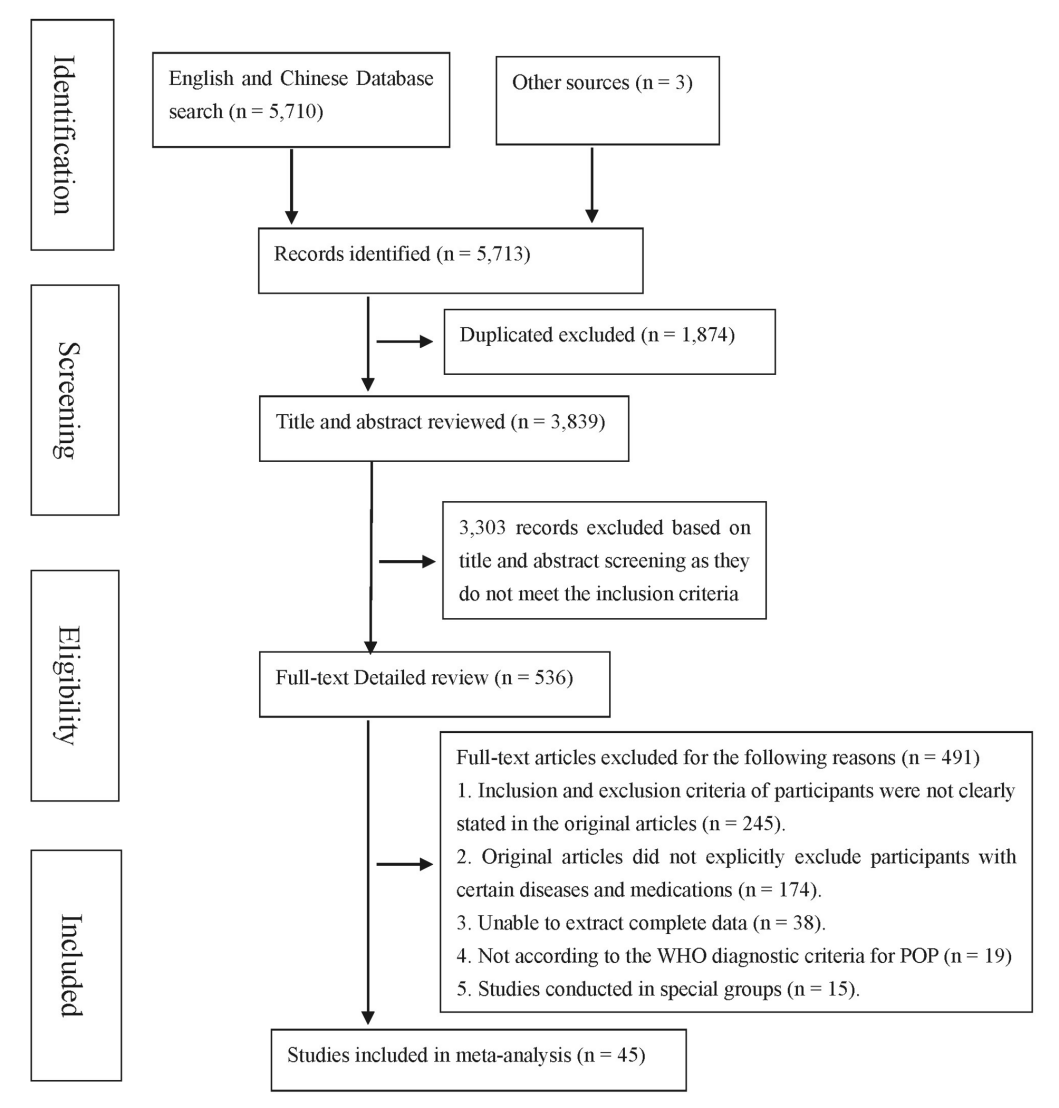
Flow diagram of included/excluded studies

The literature screening process and its outcome are shown in Fig. 1. In brief, 5,713 articles were initially identified, and 1,874 articles were removed for duplication. 3,303 unrelated articles were further excluded after title and abstract screening. After reviewing 536 full-text articles with detailed reading, finally, leaving 45 articles for final analyses.

### Characteristics of included studies

Characteristics of the included 45 studies [21–65] are shown in Additional file 1. These studies were published between 2002 and 2023, while data were collected from 1997 to 2021. Among these, 25 studies were conducted in southern China [21–25, 29, 30, 33, 38, 41, 42, 44, 45, 47–49, 51–53, 55, 56, 59–61, 64], 19 studies were in northern China [26–28, 31, 32, 34–37, 39, 40, 46, 50, 54, 57, 58, 62, 63, 65], and only one study was conducted nationwide [43]. Moreover, a total of 23 studies reported the prevalence of the entire population (both men and women) over the age of 20 years [21–23, 26–29, 33, 35–39, 44, 47, 49, 52, 54, 58–60, 62, 63]. These 23 studies were analysed for overall data as well as subgroups (period of data collection, gender, area, BMD measurement positions) prevalence. Nineteen studies reported prevalence only of the middle-aged or older population (aged ≥ 40 years) and were thus used for age-specific group analyses [24, 25, 30, 31, 34, 40–42, 45, 46, 48, 50, 51, 55–57, 61, 64, 65]. Only two studies exclusively reported the prevalence rate among men [32, 65], while seven studies focused on postmenopausal women [25, 30, 40, 48, 55, 57, 61].

The sample size per study ranged from 151 to 75,321 creating a total population of 241,813 participants for this meta-analysis, including 113,613 men and 128,200 women. The point prevalence of POP varied between 1.1% and 63.1%. All studies had a cross-sectional design and all response rates for the survey were above 90%.

### Risk of bias in included studies

According to the Agency for Healthcare Research and Quality (AHRQ), among the 45 studies included, nine were categorized as high-quality, while 29 were deemed moderate-quality cross-sectional studies. Additionally, seven studies scored less than five points, indicating low quality. Overall, 84.44% of the included studies demonstrated acceptable quality, falling within the moderate or high categories.

### Pooled prevalence rates of osteoporosis

#### Overall

**Fig. 2:**
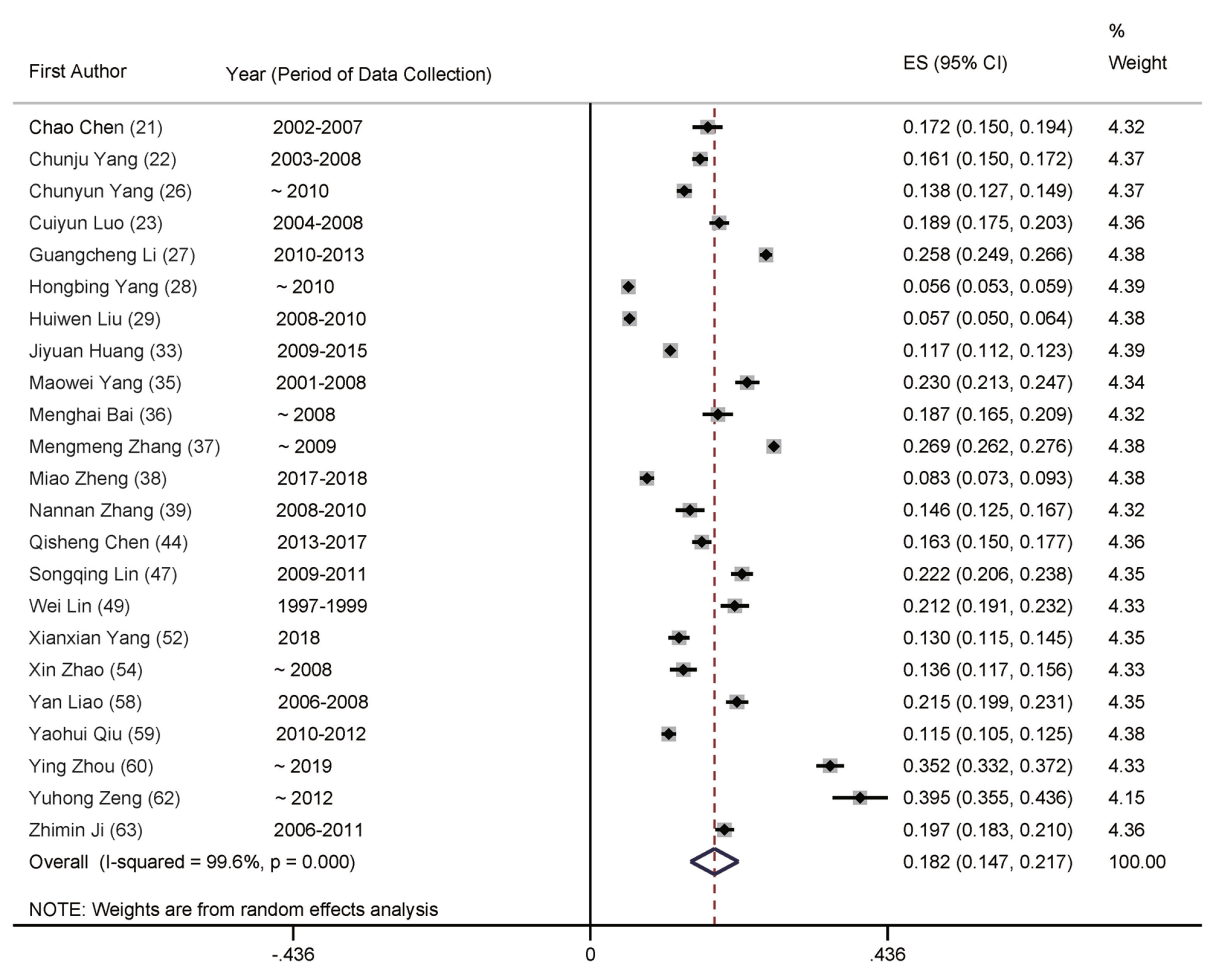
Forest plot of prevalence of primary osteoporosis for total population

**Fig. 3:**
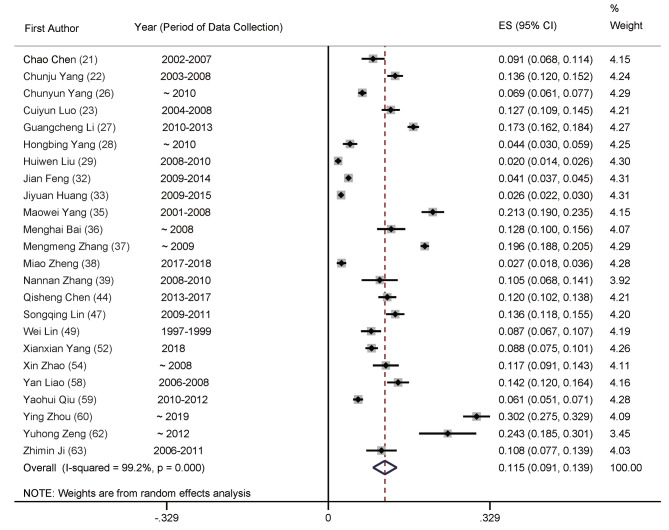
Forest plot of prevalence of primary osteoporosis for men

**Fig. 4:**
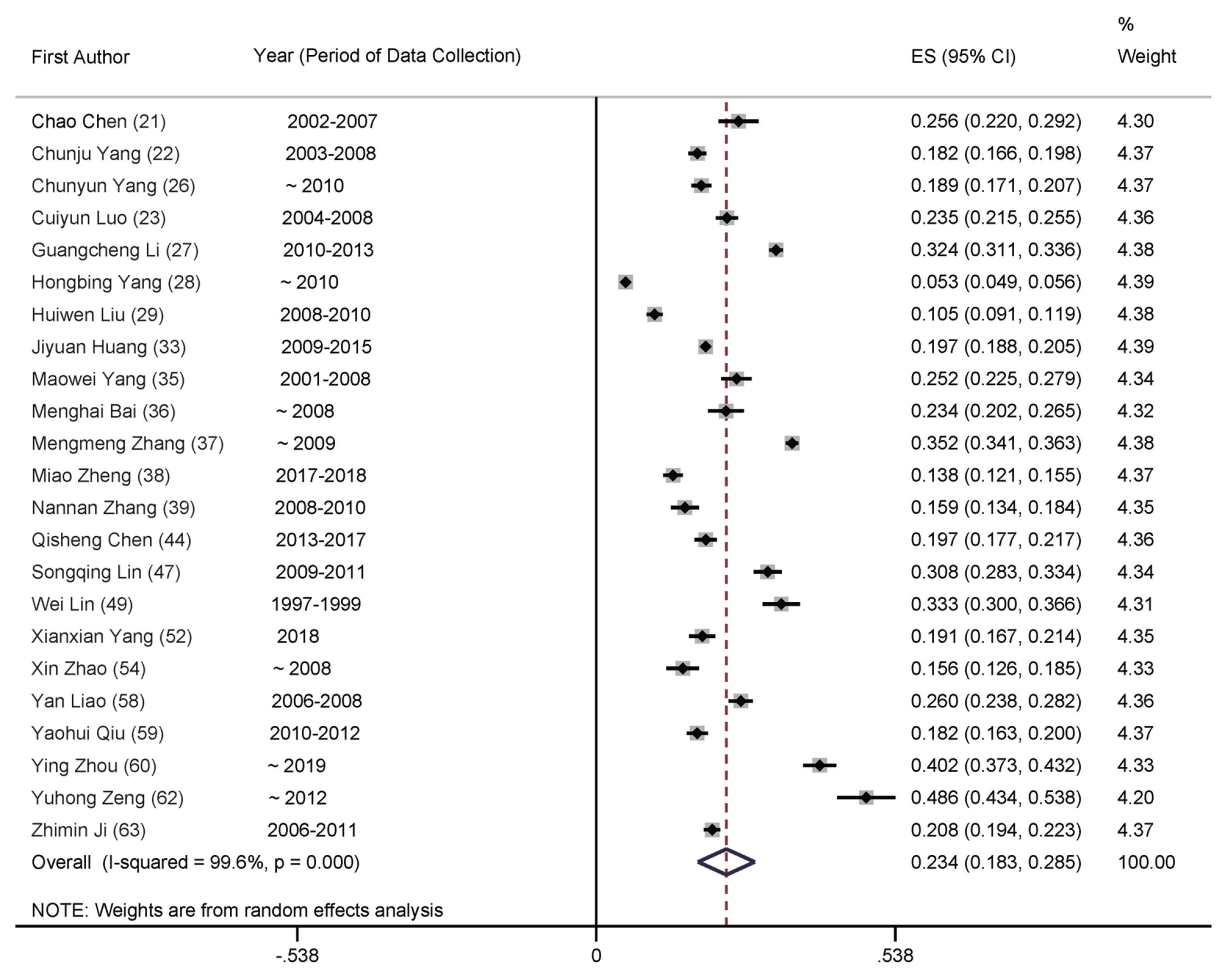
Forest plot of prevalence of primary osteoporosis for women

The meta-analysis of total prevalence estimates of studies with participants aged ≥ 20 years (*n* = 23, *N* = 106,725) revealed the prevalence rate of POP in China at 18.2% (95% CI: 14.7–21.7%, Fig. 2), with a high level of heterogeneity (99.6%). Numbers for men were *n* = 24 (*N* = 53,746), with 11.5% (95% CI: 9.1–13.9%, Fig. 3) and women were *n* = 23 (*N* = 61,879) at 23.4% (95% CI: 18.3–28.5%, Fig. 4), with significant differences between them.

#### Period of data collection

Period of Data Collection was divided into two groups: before 2010 with the 13 studies and 2011–2020 with eight studies. Meta-analysis results indicated a general upward trend (see Additional file 2). The prevalence rates from studies with data before 2010, and from 2011 to 2020 were 16.9% (95%CI: 11.9–21.8%) and 20.3% (15.1–25.4%), respectively. These data indicated that POP prevalence has increased over the past decade.

#### Area

The prevalence rate of POP in southern China was 16.4% (95% CI: 12.9–19.9%), which was lower than that in northern China 20.2% (13.4–21.0%) (see Additional file 2).

#### BMD measurement positions

Most studies measured BMD of lumbar spine, femoral neck, Ward’s triangle and greater trochanter, while only a few studies measured other sites (forearm distal 1/3 of ulna and radius). However, a subgroup analyses of included lumbar spine measurement and results from other anatomical sites revealed an almost similar prevalence rate (18.3% vs. 18.2%) (see Additional file 2).

#### Sex- and age-specific groups

Among age-specific groups, the prevalence rates of POP generally increased with age, except for the first two age groups (20–29 and 30–39 years old). Specifically, the prevalence was lowest in the 30 to 39 years age group (1.2%), while it peaked in those aged 80 years and older (53.9%). This increasing trend was also evident in other age brackets: 1.4% for 20–29 years, 4.9% for 40–49 years, 16.8% for 50–59 years, 35.2% for 60–69 years, and 44.1% for 70–79 years.

In delineating sex- and age-specific cohorts, the prevalence rates of POP exhibit a discernible consistency across genders and age brackets. Among men, prevalence rates stand at 1.6%, 1.1%, 4.2%, 9.9%, 19.0%, 27.6%, and 38.3%, respectively. Correspondingly, for women, rates are observed at 1.5%, 1.4%, 5.2%, 22.6%, 44.1%, 55.2%, and 63.1%, respectively. Notably, a prevailing trend emerges with higher prevalence rates in women compared to men across most age strata, with the exception noted in the 20–29 age group (see Additional file 2).

### Heterogeneity analysis: meta-regression analysis

When assessed collectively, a notable degree of heterogeneity was evident among the studies, with *I*^2^ statistics ranging from 63.7 to 99.9%. An exception was observed in the age-specific group 20–29 and men aged 20–29, where the *I*² values were 8.3% and 0%, respectively (see Additional file 2). Meta-regression analysis (Table 1) revealed that the overall prevalence estimates remained largely unaffected by the period of data collection, geographical area, or BMD measurement positions. However, sample size was found to exert a significant impact on the point prevalence estimate (*p* = 0.037).

**Table 1:** Results of meta-regression for prevalence of primary osteoporosis

Covariate	Meta-regression coefficient	95% Confidence interval	Adj *R*-squared (%)	*p* value
Period of Data Collection (∼ 2010 vs. 2011–2020)	0.1396	-0.3518–0.6311	3.58	0.559
Area (southern vs. northern)	0.1912	-0.2419–0.6243	1.48	0.369
Sample size, continuous	-0.4362	-0.843 – -0.0293	13.13	0.037*
BMD measurement positions (Included lumbar spine vs. other)	-0.0884	-0.4003– 0.5771	3.48	0.713

***Note***: * Significant at *p* < 0.05 (2-tailed)

The results of Meta-regression still not fully explain the high level of heterogeneity observed. Alternatively, high heterogeneity may be caused by multiple factors (e.g., sample size, measuring instrument, etc.) with synergistic effects.

### Sensitivity analysis

**Fig. 5:**
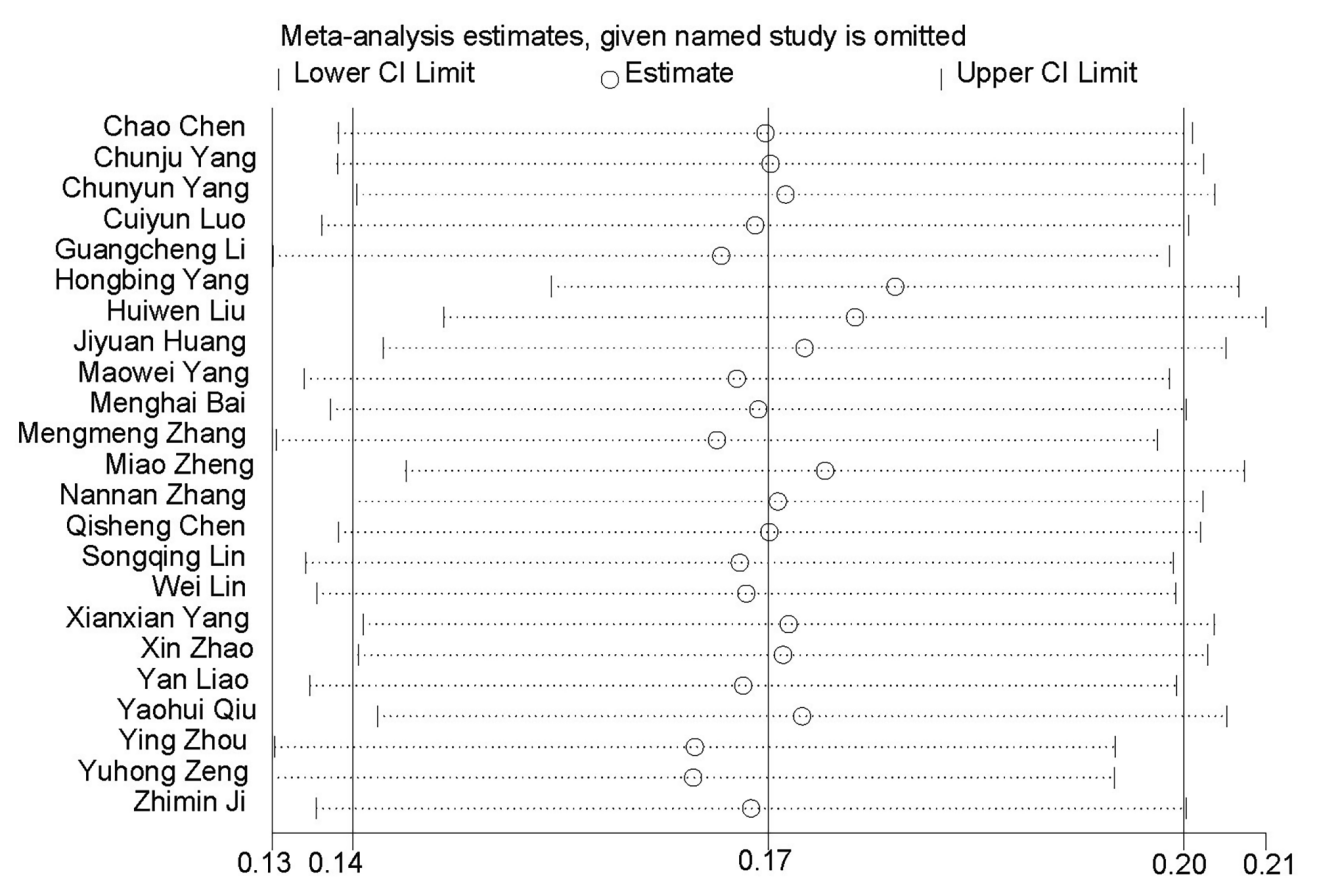
Sensitivity analysis

A sensitivity analysis was conducted by systematically excluding each study and re-evaluating the merged statistics. No significant directional changes were noted, indicating the stability of the results (Fig. 5).

### Publication bias

**Fig. 6:**
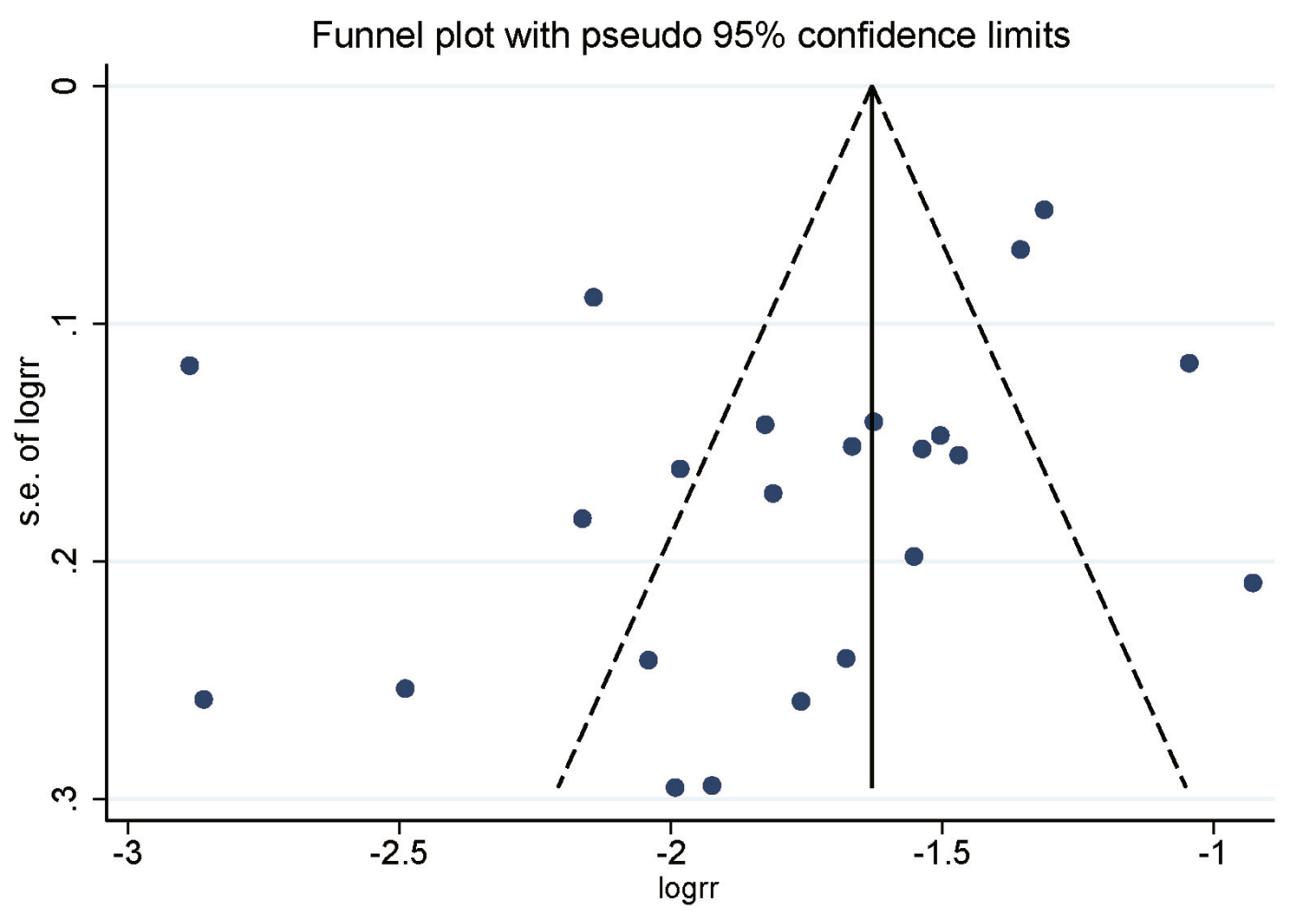
Funnel plot for publication bias

No evidence of asymmetry was observed in the funnel plot constructed using data from the 23 studies with participants over the age of 20 (Fig. 6). However, the Begg test (*p* = 0.086) and Egger test (*p* = 0.058) suggested a low likelihood of publication bias, although exceptions were noted for certain subgroups (see Additional file 2). The region parameter (urban or rural) was not included as a subgroup in this meta-analysis due to the lack of reporting on participants’ regions of residence in most studies. Additionally, as many studies reported a prevalence of zero for men and women aged 20 to 29 years, it was not possible to calculate publication bias for this age group.

## Discussion

This study conducted a systematic review and meta-analysis to determine the prevalence of POP across all age groups of Chinese adults (included young adults, postmenopausal women, middle-aged, and older individuals). The differences of prevalence among different years of data collection, genders, areas and ages were also analysed. Accurate prevalence data assumes paramount significance in fortifying the healthcare system’s efficacy in managing conditions like POP. Such data serves as a cornerstone in devising preventive strategies, formulating robust control measures, and informing strategic decision-making and policy initiatives. In recent years, numerous studies have meticulously investigated and consolidated the prevalence of osteoporosis in China.

This study identified several characteristics of POP in China. Firstly, a meta-analysis involving 106,725 subjects aged over 20 years was conducted. The findings revealed that the overall prevalence of POP in China over the past two decades stood at 18.2%, slightly lower than the global osteoporosis prevalence rate of 19.7% [66]. Notably, women exhibited a significantly higher prevalence rate (23.4%) compared to men (11.5%). Furthermore, the prevalence rate among women surpassed that of men across all age groups except for the 20–29 age bracket. This gender discrepancy can be attributed to men generally experiencing a slower rate of BMD loss [6], while estrogen deficiency in postmenopausal women can accelerate bone loss, contributing to their higher prevalence rate.

Secondly, there has been a discernible rise in the prevalence of POP over the past decade, with rates escalating from 16.9 to 20.3%. This observed trend aligns with the findings of Chen et al. [14], who documented a pronounced increase in osteoporosis prevalence in China from 2008 to 2015. However, the specific drivers behind this upward trajectory were not elucidated by Chen et al. The primary catalyst for this phenomenon is likely the demographic shift towards an aging population [5]. Furthermore, it is conceivable that the surge in POP prevalence is intricately linked to contemporary lifestyle modifications, such as inadequate physical activity, suboptimal calcium intake, and excessive emphasis on weight reduction [67]. Additionally, detrimental modern lifestyle practices, including smoking and excessive alcohol consumption, are well-established risk factors for osteoporosis [68]. Moreover, hormonal imbalances, particularly in postmenopausal women attributable to declining estrogen levels, precipitate accelerated bone resorption and heighten the likelihood of POP.

Thirdly, the prevalence of POP in southern regions is notably lower compared to northern areas. This finding directly contradicts the conclusions drawn by Chen et al. [14], highlighting discrepancies in regional trends of POP prevalence. Some contributing factors may include variations in lifestyle, diet, environmental conditions, and genetics. Notably, the warm and humid climate of southern regions facilitates outdoor physical activities, which can enhance bone density and help prevent osteoporosis [43].

Fourthly, the prevalence of POP observed in this study among the older population was notably elevated, particularly among older women (60–69: 35.2%; 70–79: 44.1%; ≥80: 53.9%), consistent with previous research. Wang et al. [16] reported the age-standardized prevalence of osteoporosis at 33.5% among middle-aged and older Chinese permanent residents, with women consistently exhibiting a higher prevalence compared to men. These findings emphasize the critical importance of prioritizing prevention and management strategies for POP within the older population, especially among older women. The results from studies by He et al. 2016 [69] and Zhu et al. 2022 [15] showed that the prevalence of osteoporosis in older people over 60 years old in China was 36% and 37.7%, respectively. In this study, however, the POP prevalence in 60–69 (35.2%) and 70–79 (44.1%) age group were higher than in previous studies. In much older people aged 80 and above, the POP prevalence was as high as 53.9%, illustrating that increasing age is the most common risk factor for POP [70]. However, a survey showed that only 2.92% older patients treated with anti-osteoporosis drugs [71]. This underscores the importance of non-pharmacological interventions (e.g. Tai Chi exercise or increase consumption of food with higher calcium content) for older patients with poor medication compliance [71, 72].

Finally, the prevalence of POP across various sites for BMD measurement showed close consistency (18.2% vs. 18.3%). This observation suggests that including the forearm as a potential screening site in future osteoporosis screenings could simplify and improve the feasibility of these procedures.

China currently leads the world in the sheer number of older population [5]. Aging, an inevitable factor, emerges as a primary risk element for POP in both genders. Moreover, the incidence of POP closely correlates with modifiable lifestyle factors such as dietary imbalances, sedentary habits, insufficient exposure to sunlight, smoking, and excessive alcohol consumption [73], all of which increase the risk of developing POP. Consequently, recent studies in China have intensified the focus on evaluating the health status of this older demographic.

In recent years, China has undertaken substantial efforts to bolster public awareness and preventive measures against POP. A range of interventions has been implemented, including osteoporosis prevention education, risk factor evaluation, and the identification of high-risk groups through screening initiatives, notably exemplified by the adoption of three-step prevention programs [14]. These initiatives should be further expanded to encompass older populations and communities.

### Limitations

This meta-analysis incorporated numerous studies with substantial sample sizes, bolstered by sensitivity analysis to enhance the robustness of the findings. Nevertheless, the study acknowledges several limitations. Firstly, significant heterogeneity was observed, likely stemming from diverse factors such as variations in sample sizes. Moreover, discrepancies in the instruments utilized may contribute to clinical heterogeneity. Secondly, the inclusion criteria limited the analysis to cross-sectional studies, excluding certain original articles that lacked clarity regarding participant characteristics, particularly regarding disease status and medication use affecting bone metabolism. Despite efforts to contact original authors for additional data, such attempts yielded no further information, potentially introducing bias.

### Implications for clinical practice and future research

The evidence synthesized in this review underscores the considerable prevalence of POP within China. To advance our understanding and response to this public health challenge, several strategic recommendations emerge. Firstly, optimizing sample sizes is imperative to yield precise epidemiological insights. Initiating comprehensive surveys to ascertain the prevalence of POP, coupled with multifaceted educational campaigns, holds promise in heightening public awareness and engagement with preventive measures. Secondly, embracing a multifaceted research approach encompassing prospective, retrospective, and case-control studies is advocated. Such an approach promises to yield a nuanced understanding of the influence factors of POP, thereby furnishing critical insights for the refinement of clinical interventions. Moreover, prospective investigations or systematic review in emerging areas, such as delineating the prevalence of distinct osteoporosis subtypes (e.g., secondary or primary; senile or postmenopausal), are encouraged. These endeavors serve as indispensable epidemiological cornerstones, empowering tailored prevention and treatment strategies across the osteoporosis spectrum.

## Conclusions

In conclusion, the prevalence of POP in the Chinese population stands at 18.2%, with rates increasing gradually with age, and notably higher among females than males, particularly among postmenopausal women who are a high-risk group for POP. It is noteworthy that over the past decade, the prevalence of POP in China has seen a rise due to the surge in the older population. Therefore, urgent action is warranted in the prevention and management of POP. In addition to conventional pharmacological interventions, there is a pressing need to explore simple and feasible non-pharmacological interventions tailored to the older population or postmenopausal women. Following the recommendations of the latest guidelines, targeted exercise programs should be devised and made accessible to community residents.

### Electronic supplementary material

Below is the link to the electronic supplementary material.

Additional File 1: Basic Characteristics of Included Studies

Additional File 2: Prevalence of Primary Osteoporosis According to Different Items

## Data Availability

The datasets used and/or analysed during the current study available from the corresponding author on reasonable request.

## Abbreviations

POP: Primary Osteoporosis
PMOP: Postmenopausal Osteoporosis
BMD: Bone Mineral Density
AHRQ: Agency of Healthcare Research and Quality
REM: Random Effects Model
FEM: Fixed Effects Model
